# The “Metabolic Memory” Theory and the Early Treatment of Hyperglycemia in Prevention of Diabetic Complications

**DOI:** 10.3390/nu9050437

**Published:** 2017-04-28

**Authors:** Roberto Testa, Anna Rita Bonfigli, Francesco Prattichizzo, Lucia La Sala, Valeria De Nigris, Antonio Ceriello

**Affiliations:** 1 Experimental Models in Clinical Pathology, INRCA-IRCCS National Institute, Ancona I-60127, Italy; r.testa@inrca.it; 2 Scientific Direction, INRCA-IRCCS National Institute, Ancona I-60127, Italy; a.bonfigli@inrca.it; 3 Department of Cardiovascular and Metabolic Diseases, IRCCS Multimedica, Sesto San Giovanni I-20099, Italy; f.prattichizzo@univpm.it (F.P.); lucia.lasala@multimedica.it (L.L.S.); 4 Insititut d’Investigacions Biomèdiques August Pi i Sunyer (IDIBAPS), C/Rosselló, 149-153, Barcelona 08036, Spain; vnigris@clinic.ub.es; 5 Centro de Investigación Biomédica en Red de Diabetes y Enfermedades Metabólicas Asociadas (CIBERDEM), Barcelona 08036, Spain

**Keywords:** metabolic memory, type 2 diabetes mellitus, diabetic complications

## Abstract

Several epidemiological and prospective studies suggest that an early intensive control of hyperglycaemia is able to decrease the risk of diabetic micro- and macro-vascular complications. A growing body of experimental evidence supports the concept that the risk for diabetes complications may be linked to oxidative stress, non-enzymatic glycation of proteins, epigenetic changes, and chronic inflammation, laying the foundation for the “metabolic memory” theory. From a clinical point of view, this theory supports the need for a very early aggressive treatment, with the goal of normalizing metabolic control as soon as possible. It may also prove beneficial to introduce therapeutic agents that are able to reduce reactive species and glycation, in addition to presenting better control of glucose levels in patients with diabetes, in order to minimize long-term diabetes complications. In this review, we evaluate the effect of glucose intake and metabolism in the light of this theory.

## 1. Introduction

Diabetes mellitus is a serious illness characterized by hyperglycemia that leads to reduced life expectancy due to its specific complications. It can be controlled clinically by exogenously administering insulin, by targeting the incretin system or by using certain drugs, which increase insulin secretion, decrease glucose release from the liver, increase the use of glucose in the skeletal muscle and fat, or delay the absorption of glucose from foods. These therapies, together with improved glucose monitoring and better markers of glycemic control, allow for the maintenance of a better and tighter control of blood glucose. In spite of these improvements in therapies available for diabetes, the presence of micro- and macro-complications remains an unsolved problem. The first definition of metabolic memory came from several studies that showed that changes in microcirculation due to hyperglycaemia were relatively reversible if an early and adequate control of blood glucose was achieved. Studies conducted on a large scale [1,2,3] have shown that early intensive glycemic control decreases the risk of diabetic microvascular complications. The first study where metabolic memory was postulated was the 1987 report from Engerman et al. [4], who evaluated the extent of the arrest in the development of diabetic retinopathy derived from improved glycemic control. Later clinical trials in diabetes also provided a picture of the phenomenon called “metabolic memory” in greater detail. In the Diabetes Complications and Control Trial (DCCT), type 1 diabetic patients underwent standard or intensive treatment regimens to control their glucose levels. Data showed that the progression of microvascular complications was so profoundly reduced in patients with intensive treatment that the DCCT ended after a mean time of 6.5 years and all patients were put on intensive therapy [5]. As a follow-up to the DCCT, the Epidemiology of Diabetes Interventions and Complications (EDIC) trial, showed that patients treated with the standard treatment regimen during the DCCT still had a higher incidence of diabetic complications compared to the patients receiving intensive therapy throughout the trial several years after switching to intensive therapy [6,7]. A longer follow-up, the EDIC study, made the influence of early glycemic control on the progression to macrovascular events even more evident [1,2]. In addition, data regarding the same study have clearly demonstrated that the long-term risk of an impaired GFR (with a follow-up of 22 years) was significantly lower among subjects treated early in the course of type 1 diabetes with intensive diabetes therapy than among those treated with conventional diabetes therapy [8]. Another important clinical trial that deserves to be cited is the United Kingdom Prospective Diabetes Study (UKPDS), which emerged as important in developing the theory of “metabolic memory” in diabetes mellitus. In this trial, people who underwent intensive treatment had fewer vascular complications and fewer adverse clinical outcomes over time as compared to people who underwent standard treatment, despite showing similar HbA1c value in the long-term follow-up that ensued [3,9]. These findings suggest that early and intensive metabolic control has enduring beneficial effects in type 2 diabetes. The same conclusion was drawn from the STENO-2 study [10]. In this study, after a mean of 13.3 years (7.8 years of multifactorial intervention with tight glucose regulation and the use of renin-angiotensin system blockers, aspirin, and lipid lowering agents, and an additional 5.5 years of follow-up), a significant reduction in deaths from cardiovascular causes was seen among patients with type 2 diabetes and microalbuminuria [10]. The authors underline that the design of the study did not allow for an estimation of the exact time at which risk factors began to improve in the conventional therapy group; however, since all patients were offered an intensive treatment at the end of the trial, the improvement probably took place early during the follow-up period. This suggests that an enduring effect of early intervention, as compared with late intervention, may be a likely explanation for the continuing divergence in cardiovascular end points, rather than a simple time-to-effect relationship [10]. Furthermore, these observations support the concept that early glycemic environment is remembered, which the authors of the DCCT/EDIC study named “metabolic memory” [1]. Another interesting study was the Veterans Affairs Diabetes Trial (VADT). Although a prespecified goal of a 1.5% reduction in HbA1c levels by intensive therapy was achieved, intensive therapy with insulin for 5.6 years did not significantly decrease the risk of major cardiovascular events in poorly controlled T2DM of long-standing duration [11]. In this study, the incidence of CVD in patients who underwent an intensive therapy was significantly lower when compared to those assigned to a standard therapy over a follow-up period of 10 years. In addition, intensive glucose-lowering therapy during the VADT period was able to decrease cardiovascular events in patients with lower coronary artery calcium [12].

## 2. “Metabolic Memory”: Experimental Evidence Initially Pointed toward Oxidative Stress as Its Cause

Different mechanisms are involved in metabolic memory, including mitochondrial DNA damage, protein kinase C activation, and the polyol pathway, increased production of advanced glycation end products (AGEs), AGE receptor overexpression, increased anion superoxide formation, mitochondrial protein glycation, and hexosamine flux alterations [13]. However, targeting these mechanisms with new therapies has had limited success in slowing down the progression of diabetes complications [14]. In this paragraph, we describe some experimental evidence in vitro, from studies in animals and in humans, of the presence of metabolic memory. Initially, it was reported several years ago that “hyperglycaemic memory” possibly existed, responsible for a hyperproduction of fibronectin and collagen in endothelial cells and diabetic rats that persists after glucose normalization [15]. This “metabolic memory” has also been reported in diabetes complications verified in dogs in which retinopathy continues to progress even after hyperglycemia is corrected [4,16]. Brownlee has postulated that there is an excess of superoxide anion in the mitochondria of endothelial cells in response to hyperglycemia, which gives rise to diabetes complications [17]. Considering that this overproduction of reactive species could play a key role in the development of hyperglycemia-related diabetes complications, it is possible that the risk of complications persists even when hyperglycemia is reduced or normalized. To answer this question, different studies have investigated the effect of re-institution of good glucose control on induced hyperglycemia. Kowluru et al. demonstrated that diabetic rats with six months of poor glycemic control followed by six months of good glycemic control had no significant effect on nitrotyrosine concentration in the retina, neither on the activity of the mitochondrial antioxidant manganese superoxide dismutase, nor on the total antioxidant capacity of the tissues [18]. Similar results were found in the kidney. In rats, where the good control of glucose was initiated soon after the induction of diabetes, oxidative stress, as measured by the levels of lipid peroxides (LPOs), 8-hydroxy-2-deoxyguanosine (8-OhdG), reduced glutathione (GSH), and NO in urine and renal cortex, did not differ from those observed in normal control rats, but when re-institution of good control was delayed for six months after induction of diabetes, oxidative stress and NO remain elevated in both urine and renal cortex [19]. These data all together suggest that the damage following hyperglycemia-induced oxidative stress can be prevented when good glycemic control is initiated very early, but are not easily reversed if poor control is maintained for a longer duration.

Regarding studies on humans, we can affirm that endothelium is the main organ involved in the development of cardiovascular disease in diabetes [20]. One study indicated that endothelial dysfunction, inflammation, and oxidative stress were almost normalized after normalizing glycemia or vitamin C administration in patients with type 1 diabetes with low HbA1c levels ≤7%. Different behavior, without normalization, was observed in patients with HbA1c levels >7%, suggesting that long lasting poor glycemic control can lead to long-term endothelial dysfunction, which does not simply respond to plasma glucose normalization or to antioxidants [21,22]. In another study, oscillating glucose was found to have more deleterious effects than constant high glucose on endothelial function and oxidative stress in both controls and patients with type 2 diabetes [23]. These data are in agreement with in vitro evidence that, when endothelial cells are exposed to oscillating glucose, this environment is more deleterious than constant high glucose, which induces metabolic memory after glucose normalization [24].

## 3. Theoretical Basis for “Metabolic Memory”

As we have said before, AGEs (non-enzymatic glycation end products), glycation of mitochondrial proteins, and oxidative stress have been found to explain, at least in part, the “glycometabolic theory”. Although hyperglycemia remains a hallmark in the pathophysiology of chronic diabetes complications, it is now clear that therapies should also address a number of factors only partially related to glycemic control [25,26]. These factors appear to be related to an imbalance between oxidative stress and antioxidant capacity, which could be the link between hyperglycemia and the multiple biochemical cascades that lead to diabetes complications [27]. Two temporally-separated phases may be highlighted behind the role of AGEs in the genesis of microvascular damage. In the early years of the disease, a linear relationship between hyperglycemia, increased oxidative stress, and excessive AGE formation could be hypothesized. Later, a persistent respiratory chain protein glycation and DNA damage in the mitochondria could generate a hyperglycemia-independent vicious cycle [28], in which oxidative stress is self-supporting, and AGEs ‘feed’ this process. The effects of this metabolic imbalance could include changes in the composition and structure of the extracellular matrix, mediated by inflammatory processes induced by receptor binding of AGEs or oxidative stress [29]. Subsequent fibrosis and the extension of the extracellular structures interfere with capillary blood flow, reducing capillary density in particular [29]. These structural changes could cause endothelial dysfunction and then atherosclerosis.

Recently, epigenetic mechanisms have been hypothesized to be a crucial interface between genetic and environmental factors to explain metabolic memory [30,31,32,33]. Hyperglycaemia can induce a variety of epigenetic changes that persist for days after the normalization of glucoselevels [30,34,35,36,37], mainly through the involvement of inflammatory genes [34,35]. Among the epigenetic mechanisms studied in metabolic memory, DNA methylation and post-translational histone modifications (PTHMs) are the most extensively investigated. In particular, high glucose levels can alter the activity of PTHMs and DNA methyltransferases, with irreversible changes over time. These modifications may explain the long-term harmful effects of metabolic memory [34,35,36,37].

Additional epigenetic mechanisms have been identified. Gene expression regulation could be influenced by non-coding RNAs, including microRNAs (miRNAs), which may be the key regulators in metabolic memory modulation. More than 2000 human miRNAs have been identified to date, making them one of the most abundant classes of epigenetic regulatory molecules [38]. Initially, miRNAs were believed to act mainly as negative regulators of gene expression, by binding to the three-untranslated regions of their target protein-coding mRNAs in a sequence-dependentmanner [38]; nowadays, it is clear that they are not only post-transcriptional regulators of gene expression, but they can directly repress or stimulate target gene transcription by directly binding to promoter regions, a phenomenon called RNA activation [39]. Another interesting action of miRNAs is that they can target enzymes involved in DNA methylation and miRNA genes which, in turn, are closely regulated at the level of promoter methylation, transcription, and processing [40]. Although many studies have evaluated miRNA modulation in patients with diabetes [41], their involvement in diabetes complications has only recently been established conclusively [42,43].

Another factor which may have an important role in metabolic memory is low-grade inflammation. Inflammation plays a key role in diabetes mellitus and its vascular complications, and prolonged inflammation could mediate metabolic memory. Epigenetic mechanisms can regulate inflammatory gene expression and cardiovascular disease susceptibility—even under non-diabetic conditions—and may be accentuated by diabetes, leading to vascular complications and metabolic memory. All environmental factors that promote the development and progression of diabetes mellitus trigger an inflammatory response, promoting inflammation-mediated insulin resistance and endothelial dysfunction [31,44,45]. Necrosis factor-kappaβ (NF-κβ) plays a key role in mediating inflammatory gene expression that has been well-evaluated. Diabetic conditions can promote inflammatory gene expression via NF-κβ activation and enhance monocyte binding to endothelial and vascular smooth muscle cells, and subsequently promote monocyte-to-macrophage differentiation [31]. The NF-κβ activation induces the expression of inflammatory cytokines involved in vascular inflammation, which stimulates the generation of endothelial adhesion molecules, proteases, and other mediators [45]. The Toll-like receptor signaling is another important factor that links inflammation and oxidative stress and is a pathogenic contributor to hypertension, insulin resistance, and obesity [44]. Also, epigenetic mechanisms may activate inflammatory gene expression in vascular cells and monocytes. Gene induction by proinflammatory agents was associated with increased histone lysine acetylation in endothelial and vascular smooth musclecells [31]. These findings reinforce the idea that the mechanisms of metabolic memory may be interdependent and simultaneous. Finally, another important mechanism that leads to metabolic memory is the above cited non-enzymatic glycosylation of proteins, where Maillard reaction and other following reactions lead to the formation of AGEs, changing protein structure andfunction [46].

**Figure 1:**
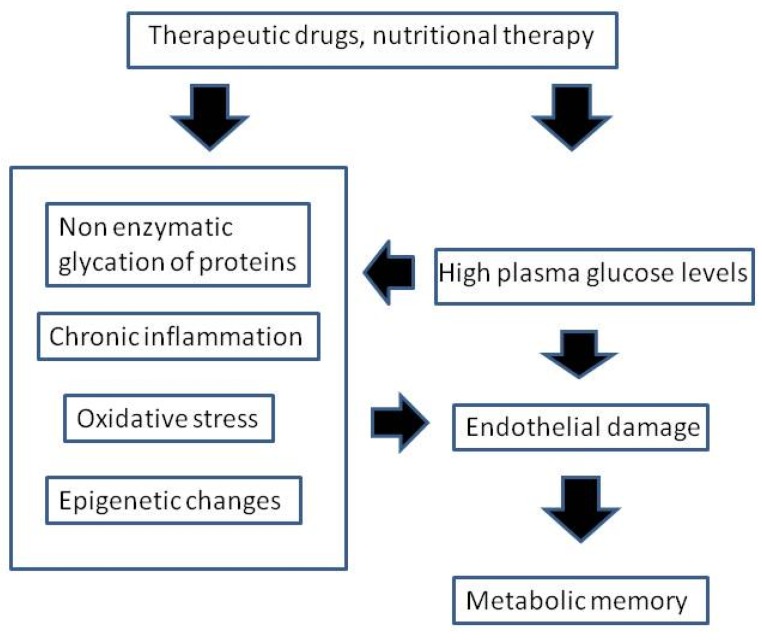
Interrelationship among high glucose levels, oxidative stress, non-enzymatic glycation of proteins, epigenetic changes, chronic inflammation, and endothelial damage.

In summary, the following four basic mechanisms have been proposed to play a role in metabolic memory: oxidative stress, non-enzymatic glycation of proteins, epigenetic changes, and chronic inflammation (Figure 1).

## 4. Therapeutic Implications and Prospects

The importance of hyperglycemia on the development of future complications has important therapeutic implications. An early aggressive treatment of this glucose imbalance becomes mandatory in patients with diabetes. This tight control should also include “postprandial” hyperglycemia [47,48] as it is accompanied by the formation of specific reactive species [49] and AGEs, not only in plasma [50] but also intracellularly [51]. Another therapeutic approach may be to attempt to reduce AGE formation, receptor of AGE (RAGE) expression, and oxidative stress generation. Different drugs have already shown the capacity to block AGE formation. Metformin and pioglitazone have been shown in vitro to prevent AGE formation [52]. ACE inhibitors and AT-1 blockers are compounds used to control blood pressure; however, they are also capable of reducing AGEs formation [53]. Telmisartan downregulates RAGE mRNA levels and subsequently inhibits superoxide generation [54], whereas gliclazide has been demonstrated to be useful in abolishing the “memory” [55]. In addition, GLP1 receptor agonists have been demonstrated to decrease inflammation, postprandial hyperlipidemia, and coagulation, resulting in a beneficial effect on atherothrombosis [56]. Interestingly, among nutritional parameters, weight loss has been shown to exert an important effect on the plasma proteome inflammation profile [57]. Meal sequence has also been demonstrated to play a role in postprandial glucose control enhancing incretin secretion [58,59]. Finally, aldose reductase inhibitors like Epalrestat have been demonstrated to protect against diabetic peripheral neuropathy by alleviating oxidative stress and inhibiting polyol pathway [60].

Meanwhile, epigenetic therapy has also been on the rise [61]. A variety of innovative pharmacological tools have been developed to target miRNA pathways [62] and to exploit miRNAs for selective gene therapy [63]. In this sense, promising in vivo results have been obtained in patients with cardiovascular disease [62,63,64]. Different experimental strategies have been evaluated to deliver miRNA mimics or antagonists. Synthetic miRNA or pre-miRNA duplexes, where an enhanced stability and a better cellular uptake was obtained with chemical modification, have been loaded onto different delivery systems—including lipid nanoparticles with surface receptor ligands—to improve tissue specificity. Adeno-associated-viral-delivery methods and other viral-based vectors have also been studied [62]. Antisense oligonucleotides, complementary to the mature miRNA sequence, or ‘antagomiRNAs’, were the first miRNA inhibitors to be used in mammals [65]. AntagomiRNAs were subjected to chemical adjustments in order to improve their pharmacokinetic and pharmacodynamic properties [61]. Finally, locked nucleic acid (LNA)-antagomiRNA technology has successfully been tested in an in vivo trial [61,62].

## 5. Conclusions

To conclude, we can affirm that hyperglycemia can cause early damage to cells of vasculature and target organs, favoring the future development of diabetes complications. This “metabolic memory” can appear even when glycemia is controlled successfully. Many questions regarding the therapeutic management of diabetes remain unanswered. Very early aggressive treatment of hyperglycemia seems to be important in the light of this “metabolic memory”.

Regarding prospective therapies, known antidiabetic drugs could be linked to new epigenetic tools.
